# Educational level-dependent melanoma awareness in a high-risk population in Switzerland

**DOI:** 10.3389/fonc.2023.1174542

**Published:** 2023-05-03

**Authors:** Alina Miriam Mueller, Elisabeth Victoria Goessinger, Sara Elisa Cerminara, Lisa Kostner, Margarida Amaral, Stephanie Marie Huber, Lea Pauline Passweg, Laura Garcia Moreno, Daniel Bodenmann, Michael Kunz, Mitchell Paul Levesque, Julia-Tatjana Maul, Phil Fang Cheng, Alexander Andreas Navarini, Lara Valeska Maul

**Affiliations:** ^1^ Department of Dermatology, University Hospital of Basel, Basel, Switzerland; ^2^ Faculty of Medicine, University of Basel, Basel, Switzerland; ^3^ Department of Dermatology, University Hospital of Zurich, Zurich, Switzerland; ^4^ Faculty of Medicine, University of Zurich, Zurich, Switzerland

**Keywords:** melanoma, awareness, prevention, sunscreen, education

## Abstract

**Introduction:**

The worldwide incidence of melanoma has been increasing rapidly in recent decades with Switzerland having one of the highest rates in Europe. Ultraviolet (UV) radiation is one of the main risk factors for skin cancer. Our objective was to investigate UV protective behavior and melanoma awareness in a high-risk cohort for melanoma.

**Methods:**

In this prospective monocentric study, we assessed general melanoma awareness and UV protection habits in at-risk patients (≥100 nevi, ≥5 dysplastic nevi, known CDKN2A mutation, and/or positive family history) and melanoma patients using questionnaires.

**Results:**

Between 01/2021 and 03/ 2022, a total of 269 patients (53.5% at-risk patients, 46.5% melanoma patients) were included. We observed a significant trend toward using a higher sun protection factor (SPF) in melanoma patients compared with at-risk patients (SPF 50+: 48% [n=60] vs. 26% [n=37]; p=0.0016). Those with a college or university degree used a high SPF significantly more often than patients with lower education levels (p=0.0007). However, higher educational levels correlated with increased annual sun exposure (p=0.041). Neither a positive family history for melanoma, nor gender or Fitzpatrick skin type influenced sun protection behavior. An age of ≥ 50 years presented as a significant risk factor for melanoma development with an odd’s ratio of 2.32. Study participation resulted in improved sun protection behavior with 51% reporting more frequent sunscreen use after study inclusion.

**Discussion:**

UV protection remains a critical factor in melanoma prevention. We suggest that melanoma awareness should continue to be raised through public skin cancer prevention campaigns with a particular focus on individuals with low levels of education.

## 1 Introduction

Worldwide, the incidence and mortality of melanoma have increased over the last decades (1). In 2020, more than 320,000 melanomas were diagnosed and about 57,000 people died from melanoma (2). Switzerland has one of the highest melanoma incidences in Europe (3). An increased exposure to ultraviolet (UV) radiation due to lifestyle changes has been recognized as a relevant factor for these trends. Besides the number of melanocytic nevi, a positive family history for melanoma and a genetic predisposition, excessive sun exposure with sunburns and the use of sunbeds significantly increases the risk of developing cutaneous melanoma (4, 5). UV radiation is thought to be responsible for 60-70% of the melanoma cases (6, 7). Both UVA (320-400 nm) and UVB (280-320 nm) play a role in photocarcinogenesis by causing direct and/or indirect DNA damage (7–10). Previous sunscreens were developed to protect against UVB, with novel products additionally incorporating UVA spectrum wavelengths (11).

Since these risk factors can be modified by sun-protective behavior, there are numerous campaigns to raise awareness among the public (12–15). Limited sun exposure during peak hours, staying in the shade, sunscreen use, wearing protective clothing and avoiding sunbeds are recommended (16). Despite these efforts, sun-protective measures are still not practiced enough in the general population and even melanoma-survivors seem to display suboptimal UV protection behavior (17, 18) despite their increased risk of developing a second melanoma or nonmelanoma skin cancer (19, 20). Skin cancer awareness seems to be linked to gender (21, 22), socio-economic class, education level (21, 23, 24) and family history of skin cancer (24).

The aim of this study was to investigate the awareness of the potential risk of UV radiation and the role of protective measures in a high-risk population for melanoma in Switzerland. We intended to identify typical characteristics that distinguish UV protective behavior in high-risk patients for melanoma that might help to further raise melanoma and sun protection awareness.

## 2 Materials and methods

### 2.1 Study design and study population

This prospective monocentric study was conducted at the Department of Dermatology of the University Hospital in Basel, Switzerland between January 2021 and March 2022. While comparing 2D- and 3D-imaging with deep-learning based risk assessments and routine skin cancer screenings conducted by dermatologists for early detection of melanoma, we assessed general melanoma awareness and UV protection habits in high-risk patients for melanoma and melanoma patients using newly created questionnaires. Patients with ≥ 100 nevi, ≥ 5 atypical nevi, a diagnosis of dysplastic nevus syndrome or a known CDKN2A mutation or a positive family history for melanoma were included in this high-risk group whereas the other group consisted of patients who had a prior diagnosis of melanoma. This division was chosen because of previously described differences in sun-protective behaviors after a melanoma diagnosis (25). Participants were recruited from ongoing consultations at our Department of Dermatology as well as referred by external primary care physicians and dermatologists. Melanoma staging was based on the 8th edition of the American Joint Committee on Cancer’s (AJCC) Cancer Staging Manual (26). Exclusion criteria were Fitzpatrick skin type IV-VI due to the current lack of compatible artificial intelligence (AI)-based risk assessment algorithm for these skin types, acute psychiatric illness, acute crisis or lack of informed consent for study participation. Written informed consent was obtained from all participants.

### 2.2 Study procedures

Based on literature research, we generated a new questionnaire consisting of 8 questions about habits regarding annual sun exposure, use of sunscreen, outdoor hobbies and sunbed use as well as the number of blistering sunburns during childhood and/or adulthood (**Supplemental Material 1**), and additionally sociodemographic data. All participants completed the survey after the standard skin cancer screening by a dermatologist and the additional 2D and 3D total body photography and AI-based risk assessments. According to the standard recommendations in skin cancer screenings, we encouraged all participants to use adequate UV protection with SPF 50+. During the 6-month and 1-year follow-up visits, participants were asked about any changes in the frequency of sunscreen application and sun protection factor (SPF). An SPF of 6-10 or 15-25 was defined as “low SPF” and 30-50 or 50+ as “high SPF”. The questionnaire consisted of multiple-choice questions with three to seven response options and binary questions (yes/no). All answers were optional.

### 2.3 Statistics

Data was analyzed using GraphPad Prism Version 9 (Graphpad Software, Inc) and RStudio (RStudio PBC). Two-sided Fisher’s exact test and chi^2^ test were used to determine associations between categorical variables. Multiple logistic regression was used to analyze the relationship between various independent variables and the occurrence of melanoma. Statistical significance was determined at an alpha level of 0.05. Missing values were listed as “unknown” in the demographics.

### 2.4 Ethics

The study was approved by the local ethics committee (22020-02482) and registered with ClinicalTrials.gov (NCT04605822). It was conducted in compliance with the Declaration of Helsinki and Good Clinical Practice rules.

## 3 Results

A total of 269 participants were included, of which 144 were at-risk patients and 125 were melanoma patients (**Table 1**). The mean age of the participants was 53.9 (+/- 14.2) years with the at-risk group being slightly younger than the melanoma group (50.9 +/- 14.5 vs. 57.3 +/- 13 years). There was an even gender distribution (52% male, 48% female). Most patients had Fitzpatrick skin type II, followed by III and I. In total, around 40% of the participants had a university degree and around 29% of both groups had a college degree. About one third of the participants (29.7%) went to secondary school or did an apprenticeship (23.6% of the at-risk patients and 36.8% of the melanoma patients). With around 50% of the cases, the most common melanoma type was superficial spreading melanoma (**Supplemental Material 2**). Melanomas on the lower extremities were most common in our population (n = 33, 26%), followed by the trunk, back and upper extremities. Most patients (44%) suspected the suspicious skin lesion themselves which has led to a medical consultation and diagnosis of a melanoma. In 32% of the cases, the presumptive diagnosis was made by a dermatologist.

**Table 1:** Patient characteristics.

Characteristics	Total	Patients at-risk formelanoma	Melanoma patients
**Number of patients (n,%)**	269	144 (53.5%)	125 (46.5%)
**Mean age (y, SD)**	53.87 (+/- 14.15)	50.92 (+/- 14.50)	57.27 (+/- 12.97)
Gender (n,%)			
Male	140 (52%)	79 (54.9%)	61 (48.8%)
Female	129 (48%)	65 (45.1%)	64 (51.2%)
Fitzpatrick skin type (n,%)			
I	15 (5.6%)	6 (4.2%)	9 (7.2%)
II	143 (53.2%)	72 (50%)	71 (56.8%)
III	111 (41.3%)	66 (45.8%)	45 (36%)
IV-VI	0	0	0
Education level (n,%)			
rimary school	2 (0.7%)	1 (0.7%)	1 (0.8%)
Secondary school and/or apprenticeship	80 (29.7%)	34 (23.6%)	46 (36.8%)
College	78 (29%)	41 (28.5%)	37 (29.6%)
University	108 (40.2%)	67 (46.5%)	41 (32.8%)
Unknown	1 (0.4%)	1 (0.7%)	0
Profession (n,%)			
Unemployed	4 (1.5%)	3 (2.1%)	1 (0.8%)
Employed/self-employed	187 (69.5%)	108 (75%)	79 (63.2%)
Retired	66 (24.5%)	28 (19.4%)	38 (30.4%)
Housewife	10 (3.7%)	4 (2.8%)	6 (4.8%)
Student	2 (0.7%)	1 (0.7%)	1 (0.8%)
Skin cancer screening frequency (n,%)			
Multiple times/year	86 (32%)	12 (8.3%)	74 (59.2%)
1x/year	81 (30.1%)	47 (32.6%)	34 (27.2%)
Every 1-2 years	24 (8.9%)	17 (11.8%)	7 (5.6%)
Every 2 years < Every 2 years	17 (6.3%) 39 (14.5%)	13 (9.1%) 35 (24.3%)	4 (3.2%) 4 (3.2%)
Never	22 (8.2%)	20 (13.9%)	2 (1.6%)

**Figure 1:**
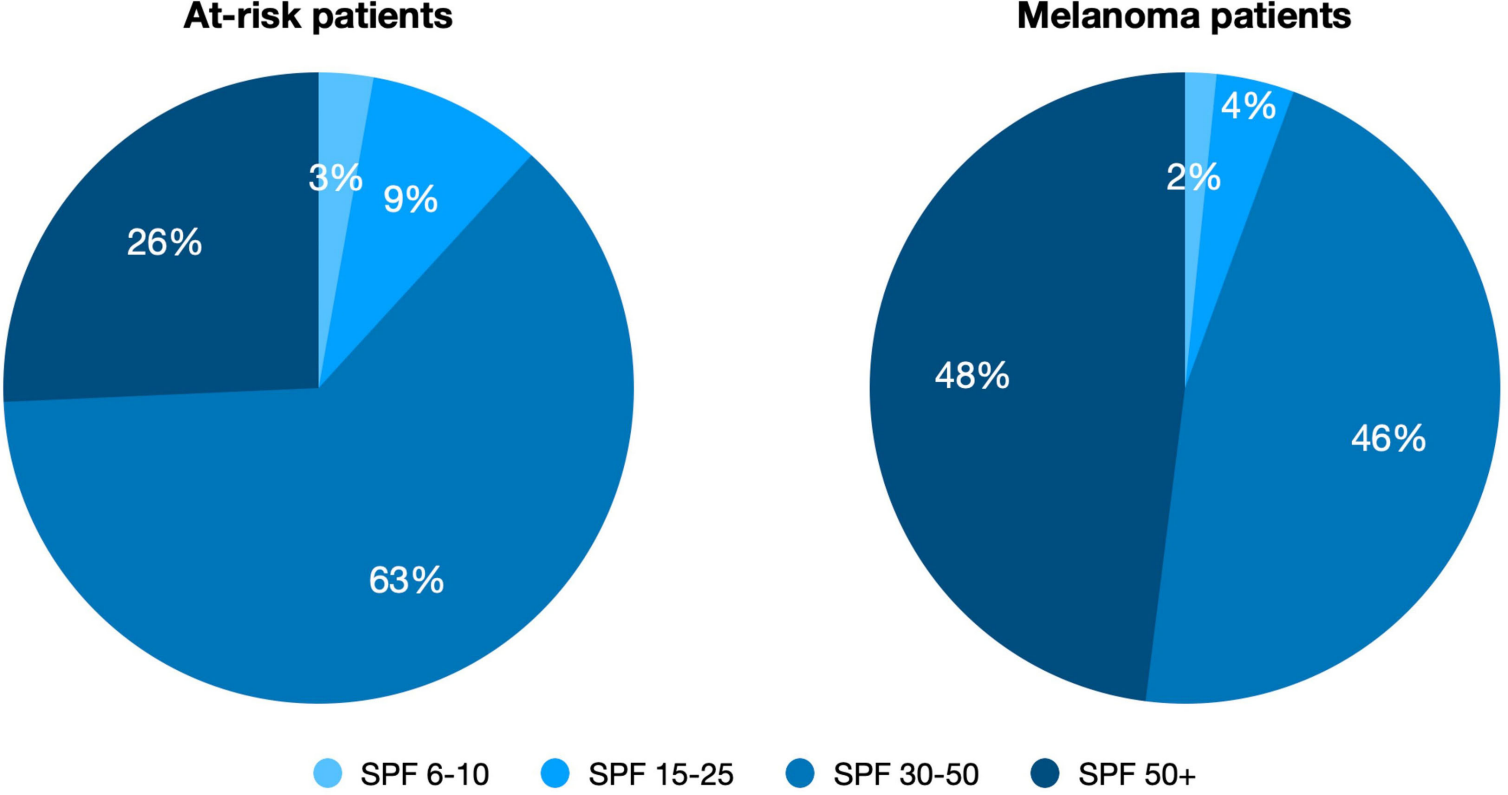
SPF use of at-risk patients vs. melanoma patients. Significant trend for melanoma patients to use higher SPF than at-risk patients (SPF 50+: 48% [n = 60] vs. 26% [n = 37], χ2 = 15.33, df = 3, p = 0.0016). SPF is color-coded.

Regarding the amount of UV exposure, sunburns, and UV protection measures, we observed that more than half of the patients (56%) reported blistering sunburns as a child whereas the majority of the participants (68%) never had blistering sunburns in adulthood (**Supplemental Material 3**). Around 27% of the melanoma patients and 32% of the at-risk patients reported using indoor tanning devices. In total, more than 90% of the participants reported applying sunscreen with a SPF of 30 or higher (**Supplemental Material 3**). We detected a significant trend for melanoma patients to use a higher SPF than at-risk patients (**Figure 1**; (SPF 50+: 48% vs. 26%, χ^2 =^ 15.33, df = 3, p = 0.0016).

**Figure 2:**
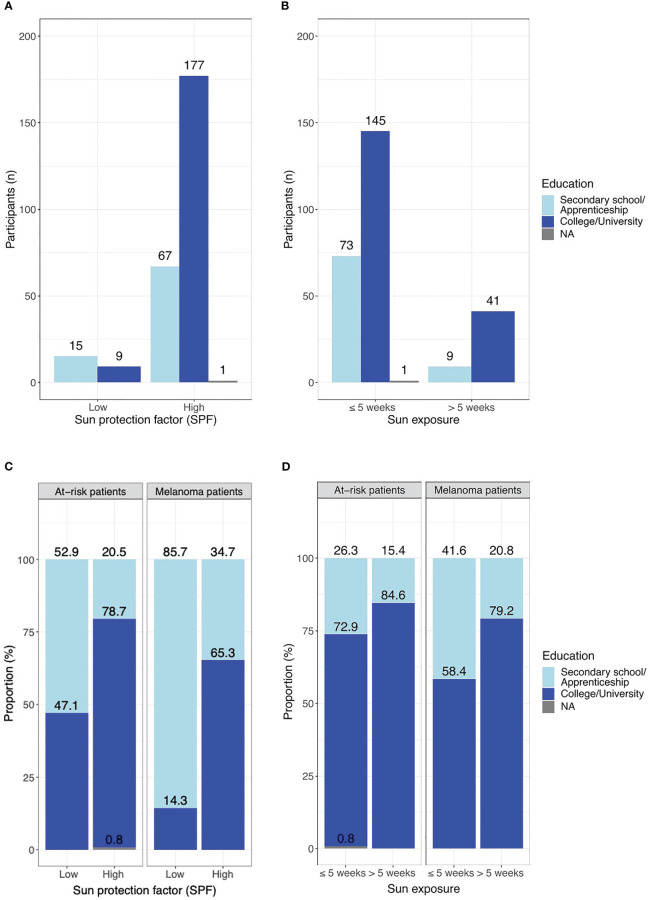
**(A)** Correlation of education level and choice of SPF. OR 4.54; 95% CI 1.92-10.83; p = 0.0007; **(B)** Correlation of education level and sun exposure per year. OR 2.23, 95% CI 1.07-4.89; p = 0.041. **(C)** Correlation of education level and choice of SPF with comparison of the at-risk cohort and melanoma patients. At-risk patients: p = 0.007; melanoma patients: p = 0.011. **(D)** Correlation of education level and sun exposure per year with comparison of the at-risk cohort and melanoma patients. at-risk patients: p = 0.2; melanoma patients: p = 0.059. Low SPF = 6-10 or 15-25, High SPF = 30-50 or 50+. Light blue = secondary school or apprenticeship, dark blue = college or university.

By investigating a possible association between education level and sun protection habits, we observed that patients who had attended college or university were significantly more likely to use a high SPF than those who only attended secondary school or did an apprenticeship (**Figure 2A**, OR 4.54; 95% CI 1.92-10.83; p = 0.0007). An opposite observation was found for annual sun exposure: Subjects with a higher education level spent significantly more time in the sun than those with a lower level (**Figure 2B**, OR 2.23, 95% CI 1.07-4.89; p = 0.041). We also performed a sub-analysis for the at-risk and melanoma patients (**Figures 2C, D**).

**Figure 3:**
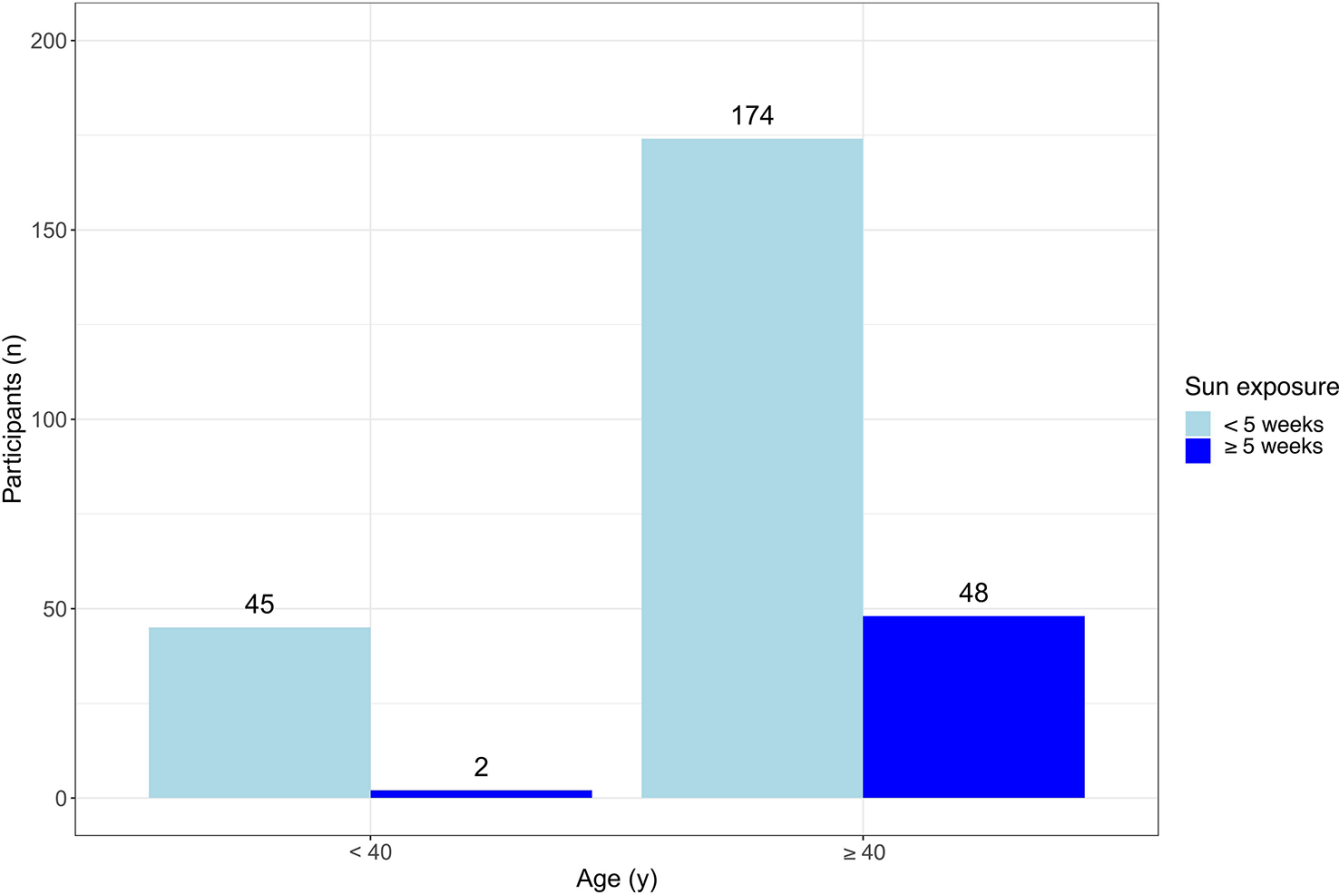
Correlation of age and sun exposure per year. Significantly more participants ≥ 40 years had a sun exposure of ≥ 5 weeks per year (OR 4.17, 95% CI 1.34-13.24; p = 0.0136). Light blue = sun exposure of not more than 5 weeks per year, dark blue = sun exposure of more than 5 weeks per year. OR 4.17, 95% CI 1.34-13.24; p = 0.0136.

Further, we detected a difference regarding different age groups and their yearly amount of sun exposure (**Figure 3**). Significantly more participants over the age of 40 years reported spending more than 5 weeks per year in the sun compared to the younger participants (**Figure 3**, OR 4.17, 95% CI 1.34-13.24; p = 0.0136). There was no statistically significant difference between different age groups and their choice of SPF.

Neither a positive family history for melanoma nor gender, Fitzpatrick skin type, melanoma subtype or stage nor the anatomic location of the melanoma on the body influenced sun protection behavior in terms of SPF used or amount of sun exposure.

At the time of this preliminary data analysis, 59 melanoma patients and 10 at-risk patients had come to their first follow-up visit. Due to the low number of cases, the results of the latter group were excluded from the current data analysis. More than half of the patients of the melanoma follow-up group (51%) reported a more frequent use of sunscreen since study participation (**Table 2**). Also, the use of sunscreen with the recommended SPF of 50+ increased within this group (**Table 2**, 47.5% vs. 57.6%).

**Table 2:** UV-protection measure changes since study participation (n = 59).

Characteristics	Melanoma patients at follow-up visit 1 (n = 59)
SPF use before study (n,%)	
6-10	1 (1.7%)
15-25	4 (6.8%)
30-50	26 (44%)
50+	28 (47.5%)
Change of sunscreen use since study participation (n,%)	
Less often	3 (5.1%)
As often	26 (44.1%)
More often	30 (50.9%)
SPF use since study participation (n,%)	
6-10	1 (1.7%)
15-25	2 (3.4%)
30-50	21 (35.6%)
50+	34 (57.6%)
Unknown	1 (1.7%)

**Figure 4:**
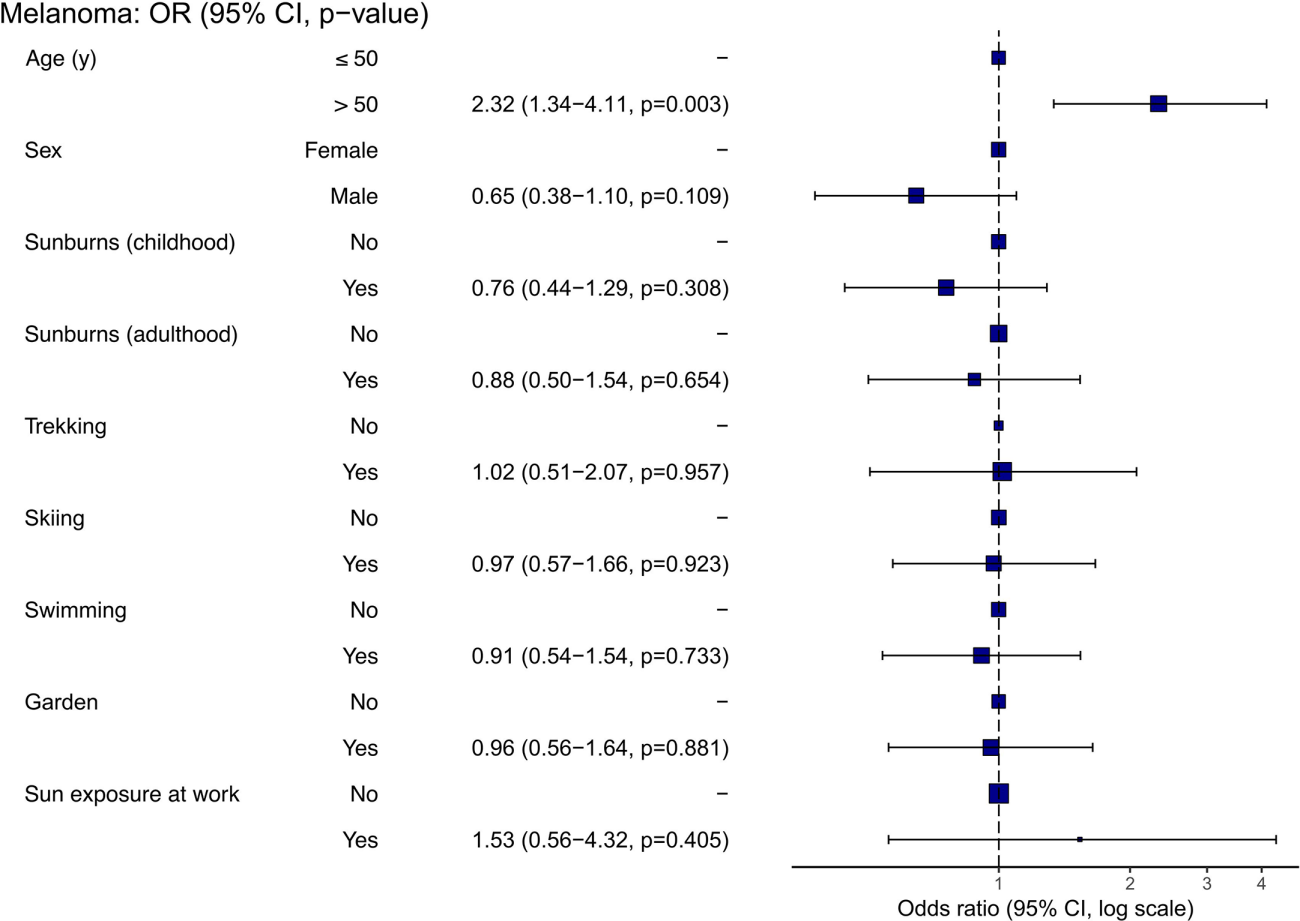
Correlations between demographics and hobbies and melanoma (data derived from melanoma patients, n = 125). An odds ratio close to 1.0 indicates that the odds are the same between the two compared groups.

To determine whether certain characteristics or behaviors were associated with cutaneous melanoma, we analyzed the data from the melanoma patient cohort and calculated the odds ratios for different variables with the endpoint of melanoma (**Figure 4**). Only an age of ≥ 50 years was significantly associated with an odds ratio of 2.32 (**Figure 4**, 95% CI 1.34-4.13, p = 0.003). Neither the occurrence of blistering sunburns during childhood or adulthood, nor sex, being exposed to the sun at work or outdoor hobbies such as trekking, skiing, swimming or gardening showed a significant association with melanoma in our cohort.

## 4 Discussion

In this prospective study, we found suboptimal sun protection behavior in both at-risk and melanoma patients. Nevertheless, we observed a trend for melanoma patients to use sunscreen with a higher SPF compared to at-risk patients. Significantly more participants with a high education level used sunscreen with a high SPF (≥ 30) than those with a lower education level, but also reported significantly more sun exposure.

At-risk and melanoma patients did not differ significantly from each other in terms of amount of sun exposure and sunburns in the past. Almost a third of the participants of both groups reported having blistering sunburns as adults despite their higher risk for melanoma occurrence. This is in line with previous studies (27, 28), displaying that a melanoma diagnosis only leads to adjusted risk behaviors in some patients. A Danish group investigated the UV exposure of melanoma patients during the first three summers after diagnosis (29) and found that after the second year, they had even more UV exposure than the healthy control group, suggesting that melanoma patients do not maintain a cautious behavior. Already in 1999, Euromelanoma was created with the aim to encourage all European countries to start skin cancer screening campaigns and to educate the population about preventive measures and treatment options (30). Despite these efforts, we still observe that even high-risk groups do not adequately implement the recommendations and skin cancer incidences continue to increase.

The observed more frequent use of sunscreen with an SPF ≥ 30 among patients with a high education level is in agreement with previously published data (21, 23, 24, 27). It could be due to a greater awareness of the possible consequences of melanoma (31) and also affects their children’s behavior (32). The knowledge about skin cancer and sun protection in childhood can already be educated in school (33, 34),

Interestingly, we observed an inverse correlation for annual sun exposure, as patients with higher education levels spent significantly more time in the sun. Researchers found that melanoma rates were 80% higher for young women living in California neighborhoods with the highest socio-economic status and UV exposure compared to the lowest (35) because of different cultural norms and/or more time to tan. Another group also found that knowledge and education level did not automatically lead to a reduction of melanoma-prone behavior, even among healthcare professionals (36). Our finding concerning increased sun exposure in older patients could have similar reasons, as they might have more resources to tan or travel. In addition, melanoma awareness campaigns have only gained momentum in the last 20 years (30), which is why they mainly impact the younger population rather than previously acquired skin damage in the elderly generation (37). We chose an age limit of 40 years for our calculations following a previous publication investigating the sun exposure profile of a French population, although interestingly, they made an opposite observation concerning sun exposure (38). In general, the correlation between age and sun protection behavior remains unclear in the literature (39).

More than one quarter of the study participants reported using sunbeds in the past. Modern sunbeds mainly emit in UVA range with a small fraction (<5%) of UVB. They can emit UV radiation 10-15 times stronger than the midday sunlight on the Mediterranean Sea (5) and are associated with a significant increase in melanoma risk (5). A recent study found that a ban on sunbeds along with a public information campaign could prevent 1206 melanoma cases, 207 melanoma deaths and 3987 non-melanoma skin cancers in the lifetime of all 18-year-olds (n=618 873) living in England in 2019 (40). Since the risk increases especially when the initial usage happens at a young age (5), Switzerland placed a ban on the use of solariums for minors in 2019 (41). In Australia and Brazil tanning salons are already prohibited completely (40, 42, 43) and recently, German and Swiss cancer leagues have been suggesting a general ban as well (44).

In contrast to previous findings (21, 22, 24, 45), we did not observe a correlation between gender, Fitzpatrick skin type or a positive family history for melanoma and sun protective behavior. We assume that a comparison of our cohorts is generally difficult, since all were patients with an increased melanoma risk who voluntarily participated in the melanoma early detection study and might have had an increased awareness primarily.

The average age of melanoma diagnosis is 57 years (46), consistent with positive correlation of melanoma and the age of ≥ 50 years in our cohort. Surprisingly, sex showed no correlation with melanoma, although according to the literature, men have a 1.5 times higher risk to develop melanoma (47). Interestingly, a history of blistering sunburns was not associated with melanoma in our study population despite data suggesting otherwise (4, 48). Workplace sun exposure also showed no correlation, which is in line with previous findings (49).

Despite observing suboptimal sun protection behavior in both at-risk and melanoma patients (21, 23–25, 27, 29, 45),, we found that compared to the general population (17), our study participants had already better sun protective behaviors before study enrollment. Nevertheless, we demonstrated that verbal education about sun protection during standard skin cancer screening by a dermatologist further improved sunscreen application frequency and SPF. The positive effects of education about sun protection by physicians, especially in high-risk populations, have been observed before (50–52). Therefore, we urge all dermatologists to instruct their patients on adequate UV protection with SPF 50+, avoidance of midday sun and wearing UV protective clothing.

The strengths of this study are a big sample size, a prospective study design in one of the European countries with the highest melanoma incidence as well as two comparative arms.

However, due to some limitations the generalizability of the results should be considered with caution. Since the questionnaires were not collected anonymously, it cannot be excluded that patients intended their answers to meet the expectations of the investigator, leading to a possible bias. Further, we were not able to obtain all the cancer characteristics due to missing data. This study focused on sun protection behavior in terms of sunscreen use. Other recommended prevention methods such as wearing protective clothing or limiting sun exposure during peak hours or staying in the shade (16) were not addressed. As only 10 participants in the at-risk group had completed their follow-up visits, they were excluded from the subanalysis concerning potential change of sun protective behavior since study participation. We plan to compare the two cohorts in this regard in a subsequent analysis after completion of the ongoing three-year prospective study. Also, future studies focusing on differences in serum vitamin D levels and oral vitamin D supplementation among different melanoma risk groups are of great interest.

UV protection remains one of the crucial factors in primary melanoma prevention. With Switzerland reporting among the highest incidence rates for melanoma in Europe, we strongly suggest to further raise melanoma awareness in public skin cancer prevention campaigns with a focus on individuals with low education levels. We propose to address the latter through campaigns on television and radio as target-oriented and promising media for this population. In addition, we suggest integrating the topic into the curriculum of schools. Further, we urge all dermatologists to instruct their patients at every examination on adequate UV protection with SPF 50+, avoidance of midday sun and wearing UV protective clothing.

## Data availability statement

The raw data supporting the conclusions of this article will be made available by the authors, without undue reservation.

## Ethics statement

The study was approved by the local ethics committee (22020- 02482) and registered with ClinicalTrials.gov (NCT04605822). The patients/participants provided their written informed consent to participate in this study.

## Author contributions

Conceptualization, LM and AM; Data curation, AM, EG, SC, LK, MA, SH, MK, LP, LG, DB, and LM; Formal analysis, AM, PC, and LM; Investigation, AM, EG, SC, LK, MA, SH, MK, and LM; Methodology, LM, J-TM, and PC; Resources, LM and AN; Writing—original draft preparation, AM and LM; Writing—review and editing, AM, EG, SC, LK, MA, SH, LP, LG, DB, MK, ML, J-TM, PC, AN, and LM; Visualization, AM, PC, and LM; Supervision, LM; Project administration, LM; Funding acquisition, LM and AN. All authors contributed to the article and approved the submitted version.
