# Supplementary material for: Educational level-dependent melanoma awareness in a high-risk population in Switzerland
Source: Front Oncol. 2023 May 3;13:1174542. doi: 10.3389/fonc.2023.1174542 (PMC10189064; doi:10.3389/fonc.2023.1174542)
Supplement: Supplementary file 1 [file Table_1.pdf]

Supplementary Material

Educational level-dependent melanoma awareness in a high-risk population in Switzerland

Alina Miriam Mueller[1,2], Elisabeth Victoria Goessinger[1,2], Sara Elisa Cerminara[1,2], Lisa Kostner[1,2], Margarida Amaral[1], Stephanie Marie Huber[1], Lea Pauline Passweg[2], Laura Garcia Moreno[2], Daniel Bodenmann[2], Michael Kunz[1], Mitchell Paul Levesque[3,4], Julia-Tatjana Maul[3,4], Phil Fang Cheng[3], Alexander Andreas Navarini[1,2] and Lara Valeska Maul[1,2,*]

*** Correspondence:**
Lara Valeska Maul
laravaleska.maul@usb.ch

Questionnaire about sun protection behavior (Baseline visit)

1. How often do you have your skin checked?
   □ several times a year
   □ every 12 months
   □ every 1-2 years
   □ every 2 years
   □ less often than every 2 years
   □ never

2. Did you have blistering sunburns as a child? □ yes □ no
   - If yes, how often?
     □ rarely (< 1x per year)
     □ regularly (1x per year)
     □ often (> 1x per year)

3. Have you had blistering sunburns as an adult? □ yes □ no
   - If yes, how often?
     □ rarely (< 1x per year)
     □ regularly (1x per year)
     □ often (> 1x per year)

4. Have you tanned in a solarium in the past? □ yes □ no

5. Do you have one or more of the following hobbies?
   - Do you hike? □ yes □ no
   - Do you ski/snowboard? □ yes □ no
   - Do you swim? □ yes □ no
   - Do you do any gardening? □ yes □ no

6. How many weeks per year are you exposed to the sun during your holidays/weekends e.g. at the sea, in the mountains, at the lake?
   □ < 1 week
   □ 1 week
   □ 2 weeks
   □ 3 weeks
   □ 4 weeks
   □ 5 weeks
   □ ≥ 6 weeks

7. Are you exposed to the sun during your working hours? □ yes □ no

8. What sun protection (SPF) do you use?
   □ SPF 6-10
   □ SPF 15-25
   □ SPF 30-50
   □ SPF 50+

Supplementary Material 1: Questionnaire about sun protection behavior.

Supplemental Material 2: Melanoma patients' characteristics (n = 125).

**Characteristics**	**Melanoma patients (n = 125)**
**AJCC stage (n,%)**	22 (17.6%)
0	81 (64.8%)
I	6 (4.8%)
II	12 (9.6%)
III	4 (3.2%)
IV-VI	0
**Melanoma subtype (n,%)**	
Lentigo maligna/In situ	20 (16%)
Lentigo maligna melanoma	7 (5.6%)
Superficial spreading melanoma	63 (50.4%)
Nodular melanoma	10 (8%)
Acrolentiginous melanoma	1 (0.8%)
Amelanotic melanoma	2 (1.6%)
Other*	11 (8.8%)
Unknown	11 (8.8%)
**Melanoma location (n,%)**	
Face/neck	18 (14.4%)
Scalp	5 (4%)
Trunk	23 (18.4%)
Back	22 (17.6%)
Upper extremities	21 (16.8%)
Lower extremities	33 (26.4%)
Unknown primarius	3 (2.4%)
**Initial suspicion of melanoma by (n,%)**	
Patient	55 (44%)
Relative	3 (2.4%)
Dermatologist	40 (32%)
General practitioner	5 (4%)
Other	22 (17.6%)

* *Nevoid melanoma, naevus bleu like melanoma, melanoma ex naevo.*

Supplemental Material 3: Sun exposure and UV protection management.

Characteristics	Total	Patients at-risk for Melanoma (n = 144)	Melanoma patients (n = 125)
**Blistering sunburns as a child (n,%)**			
> 1x/year	9 (3.4%)	6 (4.2%)	3 (2.4%)
1x/year	46 (17.1%)	27 (18.8%)	19 (15.2%)
< 1x/year	95 (35.3%)	51 (35.4%)	44 (35.2%)
Never	119 (44.2%)	60 (41.7%)	59 (47.2%)
**Blistering sunburns as an adult (n,%)**			
> 1x/year	4 (1.5%)	3 (2.1%)	1 (0.8%)
1x/year	6 (2.2%)	5 (3.5%)	1 (0.8%)
< 1x/year	77 (28.6%)	40 (27.8%)	37 (29.6%)
Never	182 (67.7%)	96 (66.7%)	86 (68.8%)
**Sunbed use (n,%)**	80 (29.7%)	46 (31.9%)	34 (27.2%)
**Outdoor hobbies (n,%)**			
Trekking	224 (83.3%)	118 (81.9%)	106 (84.8%)
Skiing	139 (51.7%)	78 (54.2%)	61 (48.8%)
Swimming	150 (55.8%)	81 (56.3%)	69 (55.2%)
Gardening	162 (60.2%)	86 (59.7%)	76 (60.8%)
**UV exposure per year (n,%)**			
< 1 week	17 (6.3%)	6 (4.2%)	11 (8.8%)
1 week	15 (5.6%)	7 (4.9%)	8 (6.4%)
2 weeks	42 (15.6%)	27 (18.8%)	15 (12%)
3 weeks	55 (20.5%)	23 (16%)	32 (22.2%)
4 weeks	56 (20.8%)	31 (21.5%)	25 (20%)
5 weeks	34 (12.6%)	24 (16.7%)	10 (8%)
≥ 6 weeks	50 (18.6%)	26 (18.1%)	24 (19.2%)
**UV exposure at work (n,%)**	19 (7.1%)	9 (6.3%)	10 (8%)
**SPF use before study participation (n,%)**			
6-10	6 (2.2%)	4 (2.8%)	2 (1.6%)
15-25	18 (6.7%)	13 (9%)	5 (4%)
30-50	148 (55%)	90 (62.5%)	58 (46.4%)
50+	97 (36.1%)	37 (25.7%)	60 (48%)
